# Genetic structuring in a Neotropical palm analyzed through an Andean orogenesis‐scenario

**DOI:** 10.1002/ece3.4216

**Published:** 2018-07-20

**Authors:** Sebastián Escobar, Jean‐Christophe Pintaud, Henrik Balslev, Rodrigo Bernal, Mónica Moraes Ramírez, Betty Millán, Rommel Montúfar

**Affiliations:** ^1^ Facultad de Ciencias Exactas y Naturales Pontificia Universidad Católica del Ecuador Quito Ecuador; ^2^ Department of Bioscience, Ecoinformatics and Biodiversity Group Aarhus University Aarhus Denmark; ^3^ DYNADIV UMR DIADE Institut de Recherche pour le Développement Montpellier France; ^4^ Instituto de Ciencias Naturales Universidad Nacional de Colombia Bogotá Colombia; ^5^ Instituto de Ecología Universidad Mayor de San Andrés La Paz Bolivia; ^6^ Museo de Historia Natural Universidad Nacional Mayor de San Marcos (UNMSM) Lima Perú

**Keywords:** genetic divergence, genetic diversity, microsatellite markers, *Oenocarpus*, phylogeography

## Abstract

Andean orogenesis has driven the development of very high plant diversity in the Neotropics through its impact on landscape evolution and climate. The analysis of the intraspecific patterns of genetic structure in plants would permit inferring the effects of Andean uplift on the evolution and diversification of Neotropical flora. In this study, using microsatellite markers and Bayesian clustering analyses, we report the presence of four genetic clusters for the palm *Oenocarpus bataua* var. *bataua* which are located within four biogeographic regions in northwestern South America: (a) Chocó rain forest, (b) Amotape‐Huancabamba Zone, (c) northwestern Amazonian rain forest, and (d) southwestern Amazonian rain forest. We hypothesize that these clusters developed following three genetic diversification events mainly promoted by Andean orogenic events. Additionally, the distinct current climate dynamics among northwestern and southwestern Amazonia may maintain the genetic diversification detected in the western Amazon basin. Genetic exchange was identified between the clusters, including across the Andes region, discarding the possibility of any cluster to diversify as a distinct intraspecific variety. We identified a hot spot of genetic diversity in the northern Peruvian Amazon around the locality of Iquitos. We also detected a decrease in diversity with distance from this area in westward and southward direction within the Amazon basin and the eastern Andean foothills. Additionally, we confirmed the existence and divergence of *O. bataua* var. *bataua* from var. *oligocarpus* in northern South America, possibly expanding the distributional range of the latter variety beyond eastern Venezuela, to the central and eastern Andean cordilleras of Colombia. Based on our results, we suggest that Andean orogenesis is the main driver of genetic structuring and diversification in *O. bataua* within northwestern South America.

## 1 INTRODUCTION

Plant diversification at the regional level is strongly linked to tectonism and subsequent climate modification (Hoorn, Wesselingh, Ter Steege, et al., 2010). The orogeny of the Andes during the Cenozoic is considered one of the most important drivers of the very high plant diversity found in the Neotropics (Antonelli, Nylander, Persson, & Sanmartín, 2009; Gentry, 1982; Hoorn, Wesselingh, Ter Steege, et al., 2010; Luebert & Weigend, 2014; Turchetto‐Zolet, Pinheiro, Salgueiro, & Palma‐Silva, 2012). Andean uplift promoted diversification in northern South America by increasing habitat heterogeneity and geographic vicariance which may promote genetic isolation and ultimately speciation (Antonelli & Sanmartín, 2011; Mora et al., 2010). Particularly, cross‐Andean vicariance (at both sides of the Andes) may have played an important role in Neotropical plant evolution as at least half of the Ecuadorian Flora could have appeared through vicariance after the Andean uplift (Balslev, 1988). Jointly, the uplift also affected regional climate by increasing rainfall along the eastern flanks of the Andes due to the disruption of large‐scale atmospheric circulation (Garreaud, Vuille, Compagnucci, & Marengo, 2009; Luebert & Weigend, 2014), resulting in the modification of the continental hydrological network (Hoorn, Wesselingh, Ter Steege, et al., 2010). As plants are responsive to past geological changes (Klimova, Hoffman, Gutierrez‐Rivera, Leon de la Luz, & Ortega‐Rubio, 2017; Meerow et al., 2015), the imprints of Andean uplift could be detected in the intraspecific genetic patterns of Neotropical plants with cross‐Andean distribution. In this sense, analyzing the genetic structure of plant species would allow inferring the effect of Andean orogenesis on their intraspecific diversification and evolution. Furthermore, this information could shed light about the generation of the highly diverse Neotropical flora.

The tropical Andes are divided into two major geographical units: central Andes and northern Andes (Weigend, 2002). The central Andes (central and southern Peru, northern Chile, and southern Bolivia) uplifted during the Paleogene (65–34 Ma), while the northern Andes (Colombia, Ecuador, and northern Peru) did so during the Neogene (23–2.6 Ma). The central and northern Andes are separated by a low transition zone known as Amotape‐Huancabamba which harbors high levels of species diversity and endemism (Borchsenius, 1997; Byg, Vormisto, & Balslev, 2006; Struwe, Haag, Heiberg, & Grant, 2009; Weigend, 2002, 2004). The Andean uplift formerly divided the former continuous rain forest into Amazonia and Chocó forests (Turchetto‐Zolet et al., 2012) and promoted the reaccommodation of adjacent foreland basins (Hoorn, Wesselingh, Ter Steege, et al., 2010). Intense mountain building occurred during the middle Miocene (12 Ma) allowing the parallel formation of a new aquatic system known as “Pebas” in the western Amazon basin (Gregory‐Wodzicki, 2000; Hoorn, Wesselingh, Hovikoski, & Guerrero, 2010; Meerow et al., 2015; Mora et al., 2010). At that time, the Andes may have started restricting gene flow between eastern and western rain forests on each side of the region, promoting vicariance processes through genetic divergence (Dick, Roubik, Gruber, & Bermingham, 2004; Trénel, Hansen, Normand, & Borchsenius, 2008). After the Pebas system disappeared and was replaced by the Amazon drainage system (10 Ma) and the establishment of the Amazon River (7 Ma), the Amazon foreland basins became overfilled by the Andean influx of water and sediments over millions of years (Hoorn, Wesselingh, Ter Steege, et al., 2010). This change in the continental hydrological system along with new events of intense tectonism during the Pliocene (5 Ma) allowed for the formation of palaeoarches in the Amazonian lowlands which promoted allopatric diversification between populations located on each side of the arches (Hoorn, Wesselingh, Ter Steege, et al., 2010; Hubert et al., 2007). The basins that formed after the uplift of the arches eventually became overfilled with Andean influx, hiding the arches underground and reconnecting Amazonian isolated biota (Espurt et al., 2010; Hubert et al., 2007).

Despite the increasing evidence that supports cross‐Andean genetic divergence in both highland and lowland Neotropical plant populations (Dick, Abdul‐Salim, & Bermingham, 2003; Dick & Heuertz, 2008; Hardesty et al., 2010; Motamayor et al., 2008) and also animals (Lovette, 2004), genetic connectivity between cross‐Andean regions has been identified (Pérez‐Escobar et al., 2017; Rymer, Dick, Vendramin, Buonamici, & Boshier, 2012; Trénel et al., 2008). Historically, biotic exchange has occurred between the Chocó and the Amazon through low passes in the Andes that have functioned as dispersal corridors. The Amotape‐Huancabamba zone in southwestern Ecuador/northwestern Peru is a region where dispersal corridors, such as the Huancabamba depression or the Girón‐Paute deflection, have facilitated historical cross‐Andean dispersal in low‐ and midelevation lineages in an east–west direction and vice versa (Quintana, Peninngton, Ulloa Ulloa, & Balslev, 2017; Weigend, 2002, 2004) during favorable climatic conditions (Haffer, 1967). Another identified dispersal corridor is Las Cruces mountain pass in the eastern cordillera of Colombia (Dick, Bermingham, Lemes, & Gribel, 2007). These dispersal corridors may have hindered diversification processes in the region by maintaining genetic connectivity between cross‐Andean regions (Trénel et al., 2008).

Besides Andean uplift, Pleistocenic climatic shifts (2.5 Ma), as explained in the theory of refugia (Haffer, 1969), were believed to be the main drivers of diversification in the region through a continuous fragmentation process of Amazonian forest. Nevertheless, its role has lately been given less emphasis as both highland and lowland organisms already diversified during Andean orogeny before the Pleistocene (Antonelli & Sanmartín, 2011; Hoorn, Wesselingh, Ter Steege, et al., 2010; Hughes & Eastwood, 2006). Modern rainfall and temperature patterns seem to be related to species richness at large time scales, but their effect on short time scales is often less evident (Antonelli & Sanmartín, 2011; Eiserhardt, Svenning, Kissling, & Balslev, 2011; Field et al., 2009). Additionally, nutrient availability also seems to be an abiotic factor that explains biodiversity accumulation (Antonelli & Sanmartín, 2011; Tuomisto, Zuquim, & Cárdenas, 2014).

*Oenocarpus bataua* is a Neotropical palm that provides a good model for exploring the structuring of genetic diversity at the regional level due to its wide distribution in northern South America (Balick, 1986), growing in several ecoregions such as the Chocó, the Amazon basin, and the Andean slopes. Despite its wide geographical distribution, its intraspecific variability has been poorly studied with only two allopatric varieties described as follows: (a) *O. bataua* var. *bataua* distributed in northwestern South America (Chocó region, Amazonia basin, and Andean slopes) from sea level to 1,400 m.a.s.l. (Henderson, 1995) and (b) *O. bataua* var. *oligocarpus* distributed in northeastern South America (Guianas, Suriname, eastern Venezuela, eastern Amazonia, and Trinidad Island) at lower altitudes up to 580 m.a.s.l. (Balick, 1986; Henderson, 1995). As it was first described by Martius in 1823, *O. bataua* has had a complex taxonomic history (Montúfar & Pintaud, 2008). Both varieties were originally described as the species *O. bataua* and *Jessenia oligocarpus* (Balick, 1986) but later included as intraspecific variation into the single species *O. bataua* (Henderson, 1995).

Here, we describe the intraspecific genetic structure of the Neotropical palm *O. bataua* in northwestern South America, and we hypothesize possible diversification scenarios linked with the evolution of the Neotropical landscape shaped by Andean orogeny and other drivers of diversification. To do so, we analyzed cross‐Andean populations of *O. bataua* var. *bataua* in Colombia, Ecuador, Peru, and Bolivia using microsatellite markers and Bayesian clustering analyses. Populations of *O. bataua* var. *oligocarpus* from French Guiana were studied only in a first step of the analysis in order to explore the genetic relationship between both varieties. We also compared the levels of genetic diversity and the inbreeding coefficients within populations to provide valuable information for its conservation and management. Finally, we propose additional research in order to improve the knowledge about the diversification and genetics of Neotropical plants. To our knowledge, this is the first work exploring the influence of Andean uplift on the regional genetic structure of a wild palm using its intraspecific genetic diversity.

## 2 MATERIALS AND METHODS

### 2.1 Study species

*Oenocarpus bataua* is an arborescent, allogamous, monoecious, and dominant Neotropical palm, reaching 26 m height with stems up to 30 cm in diameter (Dransfield et al., 2008; Henderson, 1995). It displays different habitat preferences at the regional scale. For instance, in the western Amazon basin it grows in well‐drained soils in *terra firme* forests, in the central Amazon lowlands it grows in poorly drained soils in swamp forests, and in the intermediate zone it does not show any particular habitat preference (Kahn & de Granville, 1992; Montúfar & Pintaud, 2006). *Oenocarpus bataua* flowers all year round, with peaks depending on geographic region (García, 1998; Núñez‐Avellaneda & Rojas‐Robles, 2008). Its main pollinators are beetles, but also bees, flies, and even bats may pollinate it (Barfod, Hagen, & Borchsenius, 2011). Its fruits are consumed by birds, primates, and rodents*,* with the principal long‐distance seed dispersers varying in relation to geography (Karubian, Ottewell, Link, & Di Fiore, 2015; Karubian, Sork, Roorda, Duraes, & Smith, 2010). Its fruits and the oil extracted from them are an important nutritional resource for local human populations in the Amazon basin (Balick, 1992; Balslev & Barfod, 1987; Montúfar et al., 2010; Peralta Rivero, Zonta, Moraes, & Ríos, 2008).

### 2.2 Sample collection

**Figure 1:**
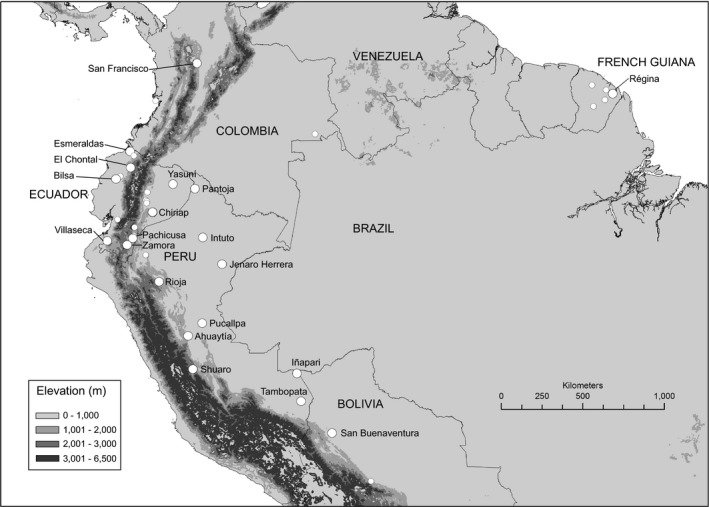
Locations of the 644 *Oenocarpus bataua* samples obtained. Individuals collected in Colombia, Ecuador, Peru, and Bolivia correspond to localities of *O. bataua* var. *bataua,* while the individuals collected in French Guiana correspond to *O. bataua* var. *oligocarpus*. The bigger circumferences represent localities with *n* > 15

Young leaves and root tissues were collected and stored in plastic bags with silica gel from a total of 644 *O. bataua* individuals at 33 localities through several sampling periods performed from August 2006 to September 2012. Five hundred and ninety‐one samples were collected from 28 localities in northwestern South America (Colombia, Ecuador, Peru, and Bolivia) and were identified as var. *bataua*; the remaining 53 samples were collected from five localities in French Guiana and identified as var. *oligocarpus* (Figure 1, Supporting Information Table S1). No samples were collected in the Brazilian Amazon region due to logistic limitations. Both adult and juvenile individuals were collected during the survey. Sampling from closely neighboring plants (distance < 15 m) was avoided to prevent temporal and full‐sibling sampling bias.

Sampling was done in a variety of ecosystems, including montane (Andes), semideciduous (southwestern Ecuador), and wet tropical lowland (Chocó and the Amazon) forests. Samples were obtained mostly from primary and secondary forests, although some sampling was done in pastures where deforestation had left few individuals standing. A particular case was the Ecuadorian Amazon locality of Chiriap (a Shuar indigenous community) where *O. bataua* samples were collected from a managed agricultural system.

### 2.3 DNA extraction, primer screening, and genotyping

Laboratory procedures were performed at IRD's (Institut de Recherche pour le Développement) Genetrop facilities in Montpellier, France. DNA extraction was done using the DNeasy plant mini kit (Qiagen) and its concentration was analyzed with a Thermo Scientific NanoDrop™ ND‐100 spectrophotometer. A total of 35 microsatellite markers (simple sequence repeats) of *O. bataua* or related (species within the tribe Euterpeae) were screened for amplification within our sampling (Gaiotto, Brondani, & Grattapaglia, 2001; Lepsch‐Cunha, Lund, & Hamilton, 2003; Montúfar, Mariac, Pham, & Pintaud, 2006). Six of these nuclear loci provided highly polymorphic SSR data and were therefore selected for further analysis (Ob02, Ob06, Ob08, Ob16, AC5‐3#4, and AG5‐5#1). One additional intron nuclear microsatellite (AG1) was used for genotyping (Ludeña et al., 2011).

SSR loci were amplified in two separate multiplex polymerase chain reactions (PCR): the first included four loci (Ob02, Ob06, Ob08, and Ob16), while the second included three (AC5‐3#4, AG5‐5#1, and AG1). Forward primers were fluorescently labeled using VIC, NED, 6‐FAM, and PET dyes. PCR amplifications were performed in a final volume of 10 μl as follows: 4 μl of DNA template (5 ng/μl), 5 μl of 2× Multiplex Mastermix, and 1 μl of 10× primer mix (containing 3 or 4 pairs of primers at 0.2 μM each primer). Thermal cycling conditions consisted of an initial denaturation step for 15 min at 96°C followed by 30 cycles of denaturation for 30 s at 94°C, annealing for 90 s at 56°C, and elongation for 90 s at 72°C. A final 10 min extension step at 72°C was added. For each PCR product, a final dilution factor of ~1/300 was obtained using 0.15 μl of size marker 500 LIZ (GeneScan™) and water. The final product of each PCR was then analyzed in a 3500 Genetic Analyzer sequencer (Applied Biosystems™), and the resulting chromatograms were examined using GeneMapper software v3.7 (Applied Biosystems™).

### 2.4 Statistical analysis

For all the sampled localities and individuals, we performed a linkage disequilibrium test for each pair of loci using the software Genepop v4.2 with default parameters (Raymond & Rousset, 1995) and also estimated the presence of null alleles with the software FreeNA (Chapuis & Estoup, 2007).

#### 2.4.1 Individual‐based analysis

In order to determine a major population structure in *O. bataua*, a Bayesian clustering method included in the software Structure v2.3 (Pritchard, Stephens, & Donnelly, 2000) was used to assign all 644 individuals to different clusters. To determine the optimal number of clusters (*K*), Structure was run under the default model of ancestry and population intercorrelation (population admixture and correlated allele frequencies) without prior information about the samples’ geographic origin. Five independent Markov chain Monte Carlo (MCMC) runs were performed using 10^4^ burn‐in generations followed by 10^5^ sampling generations, *K* ranging = 1–10. From these data, the Δ*K* statistic, developed by Evanno, Regnaut, and Goudet (2005), was computed to infer the optimal number of clusters (*K*).

To identify areas of genetic discontinuity within the distribution range of *O. bataua*, a spatial Bayesian clustering analysis was performed using Geneland software (Guillot, Mortier, & Estoup, 2005). Each individual's geo‐referenced and genotypic information was used to determine its posterior probability of belonging to a certain cluster. One MCMC run was performed using the resulting *K* value obtained from the Structure analysis (following Evanno et al., 2005) applying the following parameters: 10^5^ iterations, thinning = 1,000, allele frequencies correlated, and with uncertainty in the coordinates. Inbreeding coefficient (*F*_IS_) and differentiation values (*F*_ST_) among pairwise clusters were also obtained from Geneland. Based on the Bayesian clustering analyses, we obtained a hierarchical AMOVA in order to understand how the genetic variation is partitioned between varieties, between populations within varieties, and within populations, using the software Arlequin v3.5.2.2 (Excoffier, Laval, & Schneider, 2005) with 1,000 permutations.

We then repeated the Bayesian clustering analyses using only the samples previously identified as var. *bataua* (*n* = 566) in order to infer the population structure within this variety. Next, we used the mean *F* value computed by Structure to explore the dispersal history among genetic clusters of var. *bataua*. Under population admixture and correlated allele frequencies, the program returns an *F* value (*F*_ST_ analogue) that describes the degree of genetic differentiation of a certain cluster from a hypothetical ancestral population (Falush, Stephens, & Pritchard, 2003). The dispersal history of the clusters can be inferred as a path from low to high *F* values, assuming constant rates of genetic drift in all the clusters (Trénel et al., 2008). The mean *F* value of each cluster identified within the var. *bataua* was obtained after averaging the *F* values of the five independent MCMC with the determined optimal number of clusters (*K*). Additionally, phylogenetic relationships between clusters were depicted to determine the sequence of divergence between the clusters. A neighbor‐joining (NJ) tree was constructed in MEGA7 (Kumar, Stecher, & Tamura, 2016) using a mean matrix of allele frequency divergence among clusters (net nucleotide distance) that resulted from the analysis in Structure. The robustness of the NJ branches was evaluated using PHYLIP v3.6 (Felsenstein, 2005) through 1,000 bootstrap replications.

#### 2.4.2 Population‐based analysis

At this level, we worked with 18 localities (*n* > 15) previously identified as var. *bataua* by the first Bayesian clustering analyses, and each locality was treated as a population. In order to determine how genetic diversity was spatially distributed, allelic richness (*A*) was calculated using the rarefaction procedure implemented in FSTAT v2.9.3 (Goudet, 2001) in order to control for the effect of the different sampling size between populations. A simple linear regression between *A* and altitude from each population was performed to determine its association. Another linear regression was done between *A* and the distance from each population to the population with the highest *A* value (most diverse) to check whether genetic diversity decreased as we moved away from a center of diversity. Expected heterozygosity (*H*_e_), observed heterozygosity (*H*_o_), and the inbreeding coefficient (*F*_IS_) for each population were estimated with Arlequin.

## 3 RESULTS

Seven of the 21 pairs of loci were detected in linkage disequilibrium (*p* < 0.05; Supporting Information Table S2). All loci showed low average estimates of null allele frequency (AC5‐3#4 = 0.024, AG5‐5#1 = 0.013, AG1 = 0.022, Ob02 = 0.059, Ob06 = 0.057, Ob08 = 0.071, Ob16 = 0.068). The presence of null alleles did not affect our data as the *F*_ST_ automatically generated for all loci by the software FreeNA was similar before (0.179) and after (0.175) correction for null alleles.

### 3.1 Individual‐based analysis

**Figure 2:**
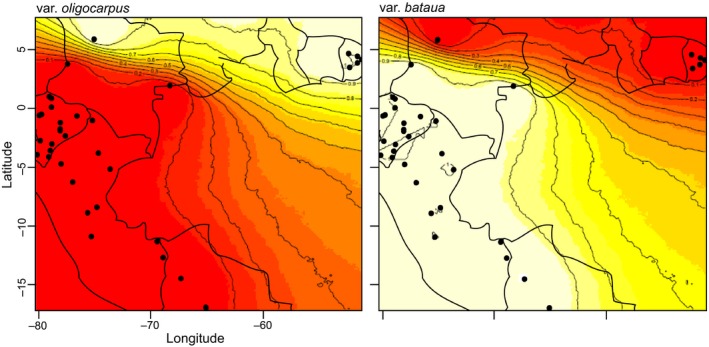
Genetic clusters identified within *Oenocarpus bataua* (var. *bataua* and var. *oligocarpus*) using 644 samples. These were identified with a spatial Bayesian clustering analysis conducted in Geneland (Guillot et al., 2005) using posterior probabilities to belong to one of *K* = 2 clusters as identified in Structure (Pritchard et al., 2000) with the statistical analysis developed by Evanno et al. (2005). Each point represents a sampled locality, while the lines represent the probability of membership to a determined cluster

Based on the Δ*K* statistic (Evanno et al., 2005), we determined the best *K* at *K* = 2 (Supporting Information Figure S1) with a clear separation between eastern (French Guiana, var. *oligocarpus*) and western (Chocó, Andean, and western Amazonian forests, var. *bataua*) populations. An exception to this pattern was the relatedness of the San Francisco population (Colombia), with the individuals identified as var. *oligocarpus* by the Bayesian analyses (Figure 2; Supporting Information Figure S2), even though it was identified in the field as var. *bataua*. Therefore, this population was not included in the following analyses on var. *bataua*. Results produced in Geneland (Figure 2) showed a strong assignment of the individuals to each corresponding cluster (var. *bataua* and var. *oligocarpus*) with posterior probabilities of membership of 1. Despite the clear divergence between the two varieties, the *F*_ST_ value obtained was moderate (Table 1). The AMOVA showed that most of the variation (73.23%) was harbored within populations, as is expected for an allogamous and long‐lived perennial species (Hamrick & Godt, 1990). Genetic variation between varieties was 13.65% and between populations within varieties was 13.12% (all *p* < 0.01**).

**Table 1:** *F*_IS_ (inbreeding coefficient) and pairwise *F*_ST_ (fixation index) values obtained from Arlequin (Excoffier et al., 2005) for the *bataua* and *oligocarpus* genetic clusters

Variety	*n*	*F*_IS_	*F*_ST_	
			*bataua*	*oligocarpus*
*bataua*	566	0.144	–	
*oligocarpus*	78	0.327	0.167	–

*Note*

*n* = sample size.

**Figure 3:**
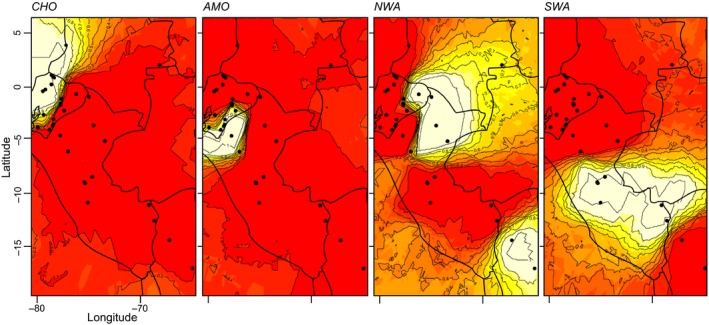
Four genetic clusters identified within *Oenocarpus bataua* var. *bataua* (*AMO*: Amotape‐Huancabamba zone; *CHO*: Chocó rain forests; *NWA*: northwestern Amazonia rain forests + northwestern Bolivia; *SWA*: southwestern Amazonia rain forests) using 566 samples. These were identified with a spatial Bayesian clustering analysis conducted in Geneland (Guillot et al., 2005) using posterior probabilities to belong to one of *K* = 4 clusters as identified in Structure (Pritchard et al., 2000) with the statistical analysis developed by Evanno et al. (2005). Each point represents a sampled locality, while the lines represent the probability of membership to a determined cluster

**Figure 4:**
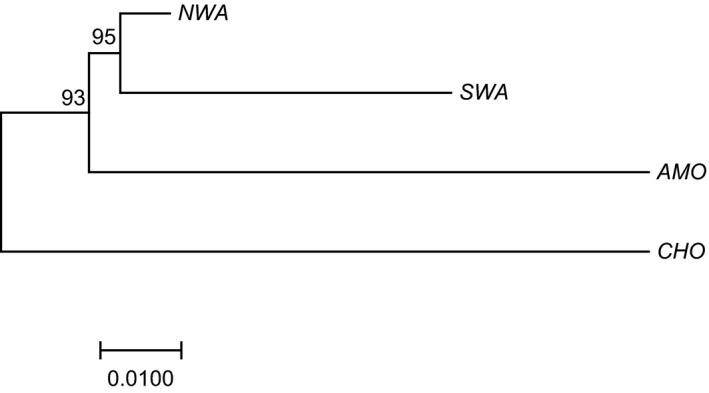
A neighbor‐joining analysis showing the phylogenetic relationships between the four genetic clusters identified within *Oenocarpus bataua* var. *bataua* (*AMO*: Amotape‐Huancabamba zone; *CHO*: Chocó rain forests; *NWA*: northwestern Amazonia rain forests + northwestern Bolivia; *SWA*: southwestern Amazonia rain forests) using 566 samples. It was performed in MEGA (Kumar et al., 2016) using a mean matrix of allele frequency divergence among clusters (net nucleotide distance) resulted from the analysis in Structure (Pritchard et al., 2000) that determined genetic clusters within *Oenocarpus bataua* var. *bataua* populations. The robustness of the neighbor‐joining branches was evaluated using PHYLIP (Felsenstein, 2005) through 1,000 bootstrap replications

Within var. *bataua*, the Structure analysis identified a peak at *K* = 4 (Supporting Information Figure S1). The bar plot generated by Structure (Supporting Information Figure S3) and the Geneland analysis (Figure 3) assigned the samples to four distinct groups that represent four regions of particular biodiversity: Chocó rain forest (hereafter *CHO*), Amotape‐Huancabamba Zone (*AMO*), northwestern Amazonian rain forest plus northwestern Bolivia (*NWA*), and southwestern Amazonian rain forest (*SWA*) (Figure 3). Based on the Structure bar plot, historical gene flow has been maintained between clusters as a great number of individuals show some probability of belonging to more than one cluster. The first cluster, *CHO,* is formed by all samples located within the Chocó rain forest. The *AMO* cluster is formed by cross‐Andean populations located in the Andean slopes of southern Ecuador/northern Peru, suggesting genetic connectivity through the Andes as the Geneland analysis (Figure 3) and the Structure bar plot (Supporting Information Figure S3) showed. The populations from the western Amazon were split among two different clusters: (a) the *NWA* cluster that included populations along the Napo River basin in Colombia, Ecuador, and Peru, and populations in northwestern Bolivia; (b) the *SWA* cluster was formed by all samples from southwestern Amazonia along the Ucayali and Madre de Dios River basins in Peru. The average *F* values obtained for the clusters in Structure suggest that the *NWA* and *SWA* clusters experienced the lowest genetic drift after the ancestral population subdivided, whereas *CHO* experienced the highest. The highest genetic differentiation (*F*_ST_ values) between clusters was found between *CHO* compared to *SWA* and *CHO* compared to *AMO*, whereas the lowest was between *NWA* and *SWA* (Table 2), showing that *O. bataua* populations within the Chocó region have diverged more than the others. These results were consistent with the phylogenetic analysis (Figure 4) in which *NWA* and *SWA* were the closest evolutionary units, and *CHO* was the most distant unit.

**Table 2:** Inbreeding coefficients (*F*_IS_) and pairwise fixation values (*F*_ST_) obtained from Geneland (Guillot et al., 2005) for the four genetic clusters identified in Structure (Pritchard et al., 2000) within *Oenocarpus bataua* var. *bataua*. Divergence estimates from a hypothetical ancestral population (*F*) were obtained from Structure (Pritchard et al., 2000)

Cluster	*F*_IS_	*F*	*F*_ST_			
			*CHO*	*AMO*	*NWA*	*SWA*
*CHO*	0.018	0.191	–			
*AMO*	0.105	0.163	0.098	–		
*NWA*	0.186	0.031	0.087	0.058	–	
*SWA*	0.097	0.053	0.134	0.091	0.050	–

*Note*

*AMO*: Amotape‐Huancabamba zone; *CHO*: Chocó rain forests; *NWA*: northwestern Amazonia rain forests + northwestern Bolivia; *SWA*: southwestern Amazonia rain forests.

### 3.2 Population‐based analysis

The lowland Amazonian populations of Intuto and Jenaro Herrera, located near Iquitos in Peru within the *NWA* cluster, had the highest genetic diversity (Table 3). Other Amazonian populations such as Pucallpa and Iñapari, located within the *SWA* cluster, also had high genetic diversity. Populations at higher elevations such as Pachicusa and Zamora, located within the cluster *AMO*, showed less diversity than those in lowland Amazonia. The lowest diversity was found in the westernmost populations within the *CHO* cluster and within Villaseca (*AMO* cluster) in southwestern Ecuador. The population of San Buenaventura in Bolivia, located at the southern limit of our sampling, showed low diversity in terms of allelic richness. No significant regression was found between *A* and altitude (*p* = 0.062) or between *A* and the distance from each population to the most diverse population (Intuto; *p* = 0.058); however, their *p* values were in the threshold of statistical significance. As all trans‐Andean (west of the Andes) populations (Esmeraldas, El Chontal, Bilsa, Villaseca) showed values of *A* under the regression line (Supporting Information Figure S4), we repeated the procedures obviating these populations. Then, a significant correlation (*p* = 0.045**) was detected between *A* and the distance from each population to Intuto, whereas no significant correlation was found between *A* and altitude (*p* = 0.054).

**Table 3:** Diversity values and inbreeding coefficients (*F*_IS_) for the 18 *Oenocarpus bataua* var. *bataua* populations with *n* > 15. Allelic richness (*A*) was calculated using the rarefaction procedure implemented in FSTAT (Goudet, 2001), whereas expected (*H*_e_) and observed (*H*_o_) heterozygosity, and the inbreeding coefficient (*F*_IS_) were obtained from Arlequin (Excoffier et al., 2005)

Locality	Cluster	*n*	*A*	*H*_e_	*H*_o_	*F*_IS_
Esmeraldas	*CHO*	16	6.191	0.70	0.81	−0.158
El Chontal	*CHO*	32	5.671	0.70	0.75	−0.074
Bilsa	*CHO*	31	5.653	0.73	0.84	−0.172
Villaseca	*CHO*/*AMO*	19	4.823	0.77	0.47	0.377^a^
Pachicusa	*AMO*	19	6.475	0.73	0.70	0.031
Zamora	*AMO*	57	6.074	0.71	0.74	−0.046
Rioja	*AMO/NWA*	18	7.718	0.76	0.61	0.157^a^
Yasuní	*NWA*	30	6.967	0.75	0.60	0.17^a^
Pantoja	*NWA*	26	7.294	0.74	0.65	0.038
Chiriap	*AMO/NWA*	30	6.917	0.72	0.88	−0.314
Intuto	*NWA*	32	9.523	0.86	0.82	0.034
Jenaro Herrera	*NWA*	30	9.371	0.81	0.86	−0.066
Pucallpa	*SWA*	43	8.072	0.76	0.75	0.007
Ahuaytía	*SWA*	32	6.385	0.67	0.66	0.01
Shuaro	*SWA*	15	7.054	0.71	0.53	0.242^a^
Iñapari	*SWA*	30	7.848	0.77	0.82	−0.064
Tambopata	*SWA*	35	6.952	0.72	0.68	0.045
San Buenaventura	*NWA*	17	5.806	0.75	0.60	0.23^a^

*Notes*

*AMO*: Amotape‐Huancabamba zone; *CHO*: Chocó rain forests; *NWA*: northwestern Amazonia rain forests + northwestern Bolivia; *SWA*: southwestern Amazonia rain forests.

*n* = sample size.

a Highly significant (*p* < 0.01).

The inbreeding coefficient tended to be low with few exceptions (Table 3). The highest *F*_IS_ value was observed in Villaseca. High and significant *F*_IS_ values were also found in the Andean populations Shuaro and Rioja in Peru and for the Amazonian population of San Buenaventura in Bolivia, where deforestation processes have occurred recently and were observed during sampling. An unexpected moderate inbreeding coefficient was observed in Yasuní in western Ecuador, located within a protected national park. A particular case with excess of heterozygosity came from Chiriap in Ecuador.

## 4 DISCUSSION

### 4.1 Andean uplift as possible driver of divergence

The four genetic clusters identified in the Bayesian analysis within var. *bataua* (Figure 3) correlated to major ecoregions recognized within northwestern South America (Dinerstein et al., 1995; Olson & Dinerstein, 2002; Weigend, 2002). We hypothesize that Andean uplift promoted three events of diversification that shaped the genetic structure of var. *bataua* into four clusters. Despite not being able to prove this hypothesis with our data, we will explore possible orogenic scenarios that can explain the divergence observed. We suggest that a first diversification event in var. *bataua* occurred between the Chocó region and the Amazon basin. Cross‐Andean divergence has been also reported for several rain forest trees in the Neotropics (Dick & Heuertz, 2008; Dick et al., 2003, 2007; Hardesty et al., 2010; Motamayor et al., 2008; Rymer et al., 2012). Although this seems like a logical explanation, we cannot discard the possibility that the cross‐Andean distribution of *O. bataua* may be due to long‐distance dispersal processes after the Andes reached its current height just 2.7 Ma (Gregory‐Wodzicki, 2000; Mora et al., 2010). In this sense, a dated phylogeny of *O. bataua* populations would help to elucidate whether this diversification event, and the other two detected, shares a time frame with the Andean orogenic events discussed or whether they happened more recently during the Pliocene.

Despite the presence of the Andes, genetic connectivity seems to occur between cross‐Andean populations in southern Ecuador/northern Peru as the Bayesian clustering analyses showed. The disjunctive distribution of the cluster *AMO* suggested that cross‐Andean populations within the Amotape‐Huancabamba zone have maintained genetic connectivity across the Andes through dispersal corridors. This cluster may have developed due to a second diversification process influenced by the Andes that occurred when populations within the Amotape‐Huancabamba zone diverged from those in the Amazon basin and the Chocó region. A similar pattern of genetic structuring was reported for *Theobroma cacao* (Motamayor et al., 2008; Thomas et al., 2012), where cross‐Andean populations located in southern Ecuador were grouped into a single cluster. The same trend was reported for a clade within the genus *Macrocarpaea* in the family Gentianaceae (Struwe et al., 2009). Apparently, the Amotape‐Huancabamba zone could be an intermediate zone that receives genetic information from both trans‐ (west of the Andes) and cis‐Andean (east of the Andes) populations due to the presence of cross‐Andean dispersal corridors.

A more recent diversification process was identified in the western Amazon basin due to the presence of two distinct genetic clusters, *NWA* and *SWA*. The split between these two clusters may have occurred when the western Amazon drainage basin divided into two foreland basins (Roddaz, Viers, Brusset, Baby, & Hérail, 2005) due to the uplift of a palaeoarch known as Fitzcarrald Arch in central Peru during the Pliocene (~4 Ma; Espurt et al., 2010). This uplift, associated with Andean tectonics, created a NE–SW‐trending barrier for gene flow in the western Amazon basin that could have promoted a third diversification event influenced by Andean uplift. The geographical division of the clusters *NWA* (excluding the northwestern Bolivia section) and *SWA* correlated with the historical division of the northern and southern foreland basins. After the basins overfilled, no geographical barriers have been present in the western Amazon basin (Espurt et al., 2010); however, the intraspecific diversification between both clusters has maintained to the present. The location of these two ancient basins presents different climatic conditions currently, being northwestern Amazonia a more humid and less seasonal region than southwestern Amazonia (Silman, 2007). Therefore, current climatic dynamics may be contributing to the maintaining of intraspecific genetic diversification in *O. bataua* as palms are highly sensitive to climatic conditions (Eiserhardt et al., 2011). Variation in climate can influence flowering phenology among populations (Welt, Litt, & Franks, 2015), which may alter their gene flow patterns (Franks & Weis, 2009) and even promote reproductive isolation (Martin, Bouck, & Arnold, 2007). It is worth mentioning that the location of the clusters *NWA* and *SWA* partially correlates with the location of two Pleistocenic forest refuges (Napo and East Peruvian) proposed by Haffer (1969); however, as the theory of refugia was shown to be based on sampling artifacts (Nelson, Ferreira, da Silva, & Kawasaki, 1990), we strongly support the hypothesis that these clusters developed after geographical isolation and posterior reproductive isolation. Therefore, the determination of the flowering phenology of the populations within the clusters *NWA* and *SWA* would shed light on the influence of climatic dynamics on the genetic structuring of *O. bataua* in the western Amazon basin.

The apparent disjunct distribution of the *NWA* cluster may be a sampling artifact caused by lack of sampling in more eastern localities. It is possible that the two sections of this cluster are linked by unsampled areas in western Brazil. A similar pattern of disjunct distribution was reported for *T. cacao* (Motamayor et al., 2008), where Amazonian populations in northern Peru were genetically related to populations in southwestern Brazil.

### 4.2 Is var. oligocarpus distributed beyond eastern Venezuela?

The assignment of the San Francisco population (Magdalena River valley, between the central and eastern Andean cordilleras of Colombia) to the *oligocarpus* cluster was a surprising result. Geographically, var. *oligocarpus* is distributed in the eastern Amazon and has not been reported in northwestern South America where San Francisco is located. The particular genetic footprint of the San Francisco population is strong enough to be interpreted as a population closely related to var. *oligocarpus*. Nevertheless, other explanations could be also analyzed in following studies, such as the potential hybridization process between var. *bataua* and *oligocarpus*, or even between *O. bataua* and *O. minor*, whose hybrids have been reported in the Magdalena River valley (Núñez‐Avellaneda, 2007).

Our study confirmed the presence of two intraspecific varieties in *O. bataua* that have experienced strong genetic divergence. The geographic distribution of var. *oligocarpus* coincided with the location of the Guiana Shield, which is made up of exposed Precambrian rock that formed about 2 Ga. The geology of this area allowed for the development of a forest with a different floristic composition compared to the forests of western and central Amazonia (Gibbs & Barron, 1993). Therefore, the divergence between var. *bataua* and var. *oligocarpus* was not related to the uplift of the Andes. The molecular differentiation found between these two varieties agreed with previous studies (Montúfar, 2007; Montúfar & Pintaud, 2008). Despite the strong differentiation between the two varieties, our data were insufficient to support the hypothesis of the botanists A. Grisebach and H. Wendland, who originally described *J. oligocarpus* (var. *oligocarpus*) in 1864 as a distinctive species from *O. bataua* (Balick, 1986). The study of gene flow between the two varieties in sympatric zones (probably within the Brazilian and Venezuelan Amazon) would enhance our knowledge about the genetic patterns between them. Furthermore, the implementation of studies that determine the presence or absence of reproductive isolation (floral morphology, phenology) between the two varieties would help to cast light on their biological divergence.

### 4.3 Genetic diversity hot spot in northwestern Amazonia

Our results added evidence to a pattern of geographic correlation between high genetic diversity and high species diversity (Lankau & Strauss, 2007; Palma‐Silva et al., 2009; Vellend & Geber, 2005); however, we did not test this correlation statistically. In this study, we reported a genetic diversity hot spot of *O. bataua* in the Peruvian rain forest around Iquitos (Intuto and Jenaro Herrera populations), which spatially coincides with the high palm and tree species diversity found in these forests (Alvez‐Valles et al., 2017; Gentry, 1988; Kristiansen et al., 2011; Ter Steege et al., 2013; Valencia, Balslev, & Paz y Miño, 1994; Vormisto, Svenning, Hall, & Balslev, 2004). High genetic diversity has been also identified in cultivated cacao within this area (Motamayor et al., 2008; Sereno, Albuquerque, Vencovsky, & Figueira, 2006; Thomas et al., 2012). The high genetic diversity of *O. bataua* also was consistent with the high species diversity of the genus *Oenocarpus* in this region. The locality of La Pedrera in southeastern Colombia (~350 km from Iquitos) harbors the highest *Oenocarpus* species diversity, with six described species (Bernal, Galeano, & Henderson, 1991; Galeano & Bernal, 2010), showing that northwestern Amazonian forests and their surroundings are a center of species and genetic diversity for this genus.

Within the Amazon region, the genetic diversity of *O. bataua* peaks in the northern Peruvian Amazon and then decreases westward and southward as we move away from this area. This pattern is statistically weak when trans‐Andean populations are included in the analysis as the Andes may interfere with long‐distance dispersal events between cross‐Andean regions (Dick et al., 2004), altering the genetic patterns between regions. This gene flow constraint may explain why trans‐Andean populations harbor lower diversity than expected. This pattern of genetic diversity added evidence on the role of northwestern Amazonian rain forests as a center of diversity in *Oenocarpus*.

The high diversity harbored within northwestern Amazonia may be related to high resource availability expressed as annual rainfall and soil cation concentration (Antonelli & Sanmartín, 2011; Tuomisto et al., 2014). This region presents high rates of water availability and climate stability due to convective rain caused by the Andes even during glacial periods (Kristiansen et al., 2011; Pitman, 2000; Tuomisto et al., 2014). It also harbors higher nutrient soils than central and eastern Amazonia due to the deposition and accumulation of material eroded during Andean orogeny (Higgins et al., 2011; Hoorn, Wesselingh, Ter Steege, et al., 2010; Tuomisto et al., 2014). It is possible that diversity depends on resource availability; however, we have ignored the specific mechanisms for this.

*Oenocarpus bataua* maintained medium levels of genetic diversity within its populations when compared with other Neotropical palms genotyped with microsatellite markers. Average genetic diversity of *O. bataua* populations in terms of allelic richness (*A* = 6.93) was lower when compared with other lowland palms such as *Euterpe edulis* (*A* = 14.46; Conte, Sedrez dos Reis, Mantovani, & Vencovsky, 2008) or *Bactris gasipaes* (*A* = 7.5; Galluzzi et al., 2015). On the other hand, *O. bataua* showed higher diversity when compared with the Andean palm *Ceroxylon echinulatum* (*A* = 1.82; Trénel et al., 2008). Although there is variation in the experimental designs of the studies mentioned above, they allow inferring the levels of genetic diversity in *O. bataua* compared to other Neotropical palms.

High inbreeding coefficients were encountered in populations growing in forests under high deforestation pressures, such as Shuaro, Rioja, or San Buenaventura. A particular high inbreeding coefficient was determined for the population of Villaseca (southwestern Ecuador), located in an isolated and heavily deforested area. This population also represents the edge of the species’ ecological range, growing in semideciduous forest; therefore, its conservation would secure this ecological adaptation of *O. bataua*. In fragmented areas, the inbreeding coefficient increases along with linkage disequilibrium (Zartman, McDaniel, & Shaw, 2006). Thus, the inbreeding coefficients reported here could explain the linkage disequilibrium detected on one‐third of pairs of loci. Forest degradation and fragmentation produces high inbreeding within populations (Rhoads, Williams, & Krane, 2017); however, as the generation time of *O. bataua* may be of more than 50 years, we could not relate the observed inbreeding coefficients with recent deforestation processes. Furthermore, the high inbreeding coefficient found in the Yasuní population was also not related to deforestation as it is located within a protected area. It is possible that these populations may be under the Wahlund effect which refers to an excess of homozygotes (high *F*_IS_ values) due to population structuring (Garnier‐Géré & Chikhi, 2013); however, a detailed study is needed to confirm this. On the other hand, an excess of heterozygosity was determined in the Chiriap population in the Ecuadorian Amazon, which is managed by local indigenous people who disperse seeds of different provenances of the Amazonian forest surroundings in order to maintain a healthy population for lipid extraction; however, this value was not significant.

### 4.4 Conclusions and future perspectives

We detected three events of genetic diversification within *O. bataua* var. *bataua* that promoted the structuring of four genetic clusters in northwestern South America. In order to determine the effective role of the Andean orogenesis as a driver of intraspecific genetic diversification in the region, we encourage the realization of dated phylogenies or coalescence analyses in other Neotropical plant species with similar distribution to *O. bataua* in a similar or bigger geographical extent as this study. Based on our results, we can suggest that Andean uplift may be the main driver of genetic diversification for this palm in northwestern South America, showing the influence of this orogenic event on the evolution and diversification of Neotropical flora. As gene flow is present among the identified genetic clusters of var. *bataua*, it seems improbable that one of these clusters would diversify as a different intraspecific entity (variety) unless gene flow becomes restricted. Addressing the role of dispersal corridors in the genetic structure of widely distributed plants would provide more accurate information about the influence of the Andes in the diversification of Neotropical flora. Additional studies are necessary to understand whether populations with high inbreeding coefficients are under the Wahlund effect. Furthermore, we confirmed the existence of two varieties with strong divergence within the wide distribution of *O. bataua* whose divergence is not related to Andean orogenesis. We strongly recommend a more detailed exploring of genetic diversification in the northern Andes in Colombia and Venezuela in order to have a better understanding of the influence of the Andes within this region. Furthermore, we suggest the study of the flowering phenology of the populations within the *NWA* and *SWA* clusters to determine whether differences in climate are altering flowering times and gene flow patterns in these populations.

## AUTHOR CONTRIBUTION

R. M., J.‐C. P., and H. B. developed the study; S. E., R. M., and J.‐C. P. made the sampling; J.‐C. P., R. B., M. M. R., and B. M. provided logistics for sampling and obtained collection permits; J.‐C. P. provided logistics for laboratory work; S. E. performed laboratory work and did the statistical analyses. S. E. and R. M. wrote the manuscript; R. M., H. B., R. B., M. M. R., B. M., and J.‐C. P. corrected the manuscript.

## DATA ACCESSIBILITY

Microsatellite and geographic data are available at https://doi.org/10.5061/dryad.1r4p8.
