# Supplementary material for: Genetic structuring in a Neotropical palm analyzed through an Andean orogenesis‐scenario
Source: Ecol Evol. 2018 Jul 20;8(16):8030–42. doi: 10.1002/ece3.4216 (PMC6144996; doi:10.1002/ece3.4216)

**Supplementary material for:**

Genetic structuring in a Neotropical palm analyzed through an Andean orogenesis-scenario

S. Escobar, J.-C. Pintaud, H. Balslev, R. Bernal, M. Moraes R., B. Millán, R. Montúfar

Corresponding author: Rommel Montúfar, Departamento de Ciencias Biológicas, Pontificia Universidad Católica del Ecuador, Av. 12 de Octubre 1076 y Roca, Quito, Ecuador, [rjmontufar@puce.edu.ec](mailto:rjmontufar@puce.edu.ec)

Table S1. General information about the 33 localities where *O. bataua* individuals were sampled.

| Locality | Variety | n | Latitude | Longitude | Altitude (m) | Forest type |
| --- | --- | --- | --- | --- | --- | --- |
| San Francisco | *bataua* | 25 | 5.99336 | -75.06756 | 750 | Montane |
| Bahía Málaga | *bataua* | 1 | 3.92796 | -77.34879 | 7 | Chocoan |
| Guainía | *bataua* | 2 | 2.09109 | -68.55888 | 8 | Amazonian |
| Esmeraldas | *bataua* | 16 | 1.10 | -78.71 | 57 | Chocoan |
| El Chontal | *bataua* | 32 | 0.23419 | -78.73266 | 928 | Montane |
| Bilsa | *bataua* | 31 | -0.39386 | -79.53752 | 300 | Chocóan |
| Tinalandia | *bataua* | 9 | -0.24824 | -79.29619 | 650 | Montane |
| Tamarindo | *bataua* | 10 | -2.6238 | -79.45666 | 512 | Chocoan |
| Villaseca | *bataua* | 19 | -3.80583 | -80.01916 | 700 | Semideciduos |
| Gualaquiza | *bataua* | 6 | -3.05 | -78.50 | 1079 | Montane |
| Pachicusa | *bataua* | 19 | -3.66572 | -78.61677 | 1140 | Montane |
| Zamora | *bataua* | 57 | -4.03408 | -78.92052 | 1022 | Montane |
| Nieva | *bataua* | 7 | -4.58333 | -77.9 | 800 | Montane |
| Rioja | *bataua* | 18 | -6.06169 | -77.16831 | 840 | Montane |
| Tena | *bataua* | 6 | -1.11 | -77.80 | 500 | Amazonian |
| Yasuní | *bataua* | 30 | -0.67128 | -76.40072 | 250 | Amazonian |
| Pantoja | *bataua* | 26 | -0.95154 | -75.22696 | 190 | Amazonian |
| Puyo | *bataua* | 7 | -1.55 | -77.89 | 980 | Montane |
| Chiriap | *bataua* | 30 | -2.2331 | -77.52628 | 514 | Amazonian |
| Intuto | *bataua* | 32 | -3.62273 | -74.75322 | 145 | Amazonian |
| Jenaro Herrera | *bataua* | 30 | -5.09299 | -73.6879 | 110 | Amazonian |
| Pucallpa | *bataua* | 43 | -8.35266 | -74.78484 | 165 | Amazonian |
| Ahuaytía | *bataua* | 32 | -8.96438 | -75.62186 | 349 | Amazonian |
| Shuaro | *bataua* | 15 | -10.88333 | -75.3 | 800 | Montane |
| Iñapari | *bataua* | 30 | -11.12727 | -69.5596 | 228 | Amazonian |
| Tambopata | *bataua* | 35 | -12.58493 | -69.04568 | 194 | Amazonian |
| Carrasco | *bataua* | 6 | -17.06603 | -65.46832 | 478 | Amazonian |
| San Buenaventura | *bataua* | 17 | -14.41611 | -67.62241 | 411 | Amazonian |
| Régina | *oligocarpus* | 23 | 4.31666 | -52.13333 | 0 | Amazonian |
| Saül | *oligocarpus* | 11 | 3.61666 | -53.2 | 85 | Amazonian |
| Aratai | *oligocarpus* | 8 | 3.98333 | -52.56666 | 187 | Amazonian |
| Cacao | *oligocarpus* | 6 | 4.52779 | -52.49306 | 22 | Amazonian |
| Piste St. Élie | *oligocarpus* | 5 | 4.79636 | -53.27718 | 188 | Amazonian |

Table S2. Test of linkage disequilibrium between pairs of loci calculated with the software Genepop (Raymond & Rousset, 1995). Highly significant values (**) are not presented by Genepop.

|  | AC5-3#4 | AG5-5#1 | AG1 | Ob02 | Ob06 | Ob08 | Ob16 |
| --- | --- | --- | --- | --- | --- | --- | --- |
| AC5-3#4 | - |  |  |  |  |  |  |
| AG5-5#1 | ** | - |  |  |  |  |  |
| AG1 | ** | 0.086 | - |  |  |  |  |
| Ob02 | 0.998 | 0.649 | ** | - |  |  |  |
| Ob06 | 0.957 | 0.321 | 0.285 | ** | - |  |  |
| Ob08 | 0.201 | 0.797 | 0.974 | 0.937 | 0,451 | - |  |
| Ob16 | ** | 0,983 | 0,994 | ** | ** | 0,006 | - |

****** *p* < 0.01

Figure S1. Data from the Δ*K* statistic computed in Structure (Pritchard et al., 2000) used to infer the optimal number of clusters (*K*) as a peak in: a) *O.* *bataua* var. *bataua* and var. *oligocarpus*; b) *O.* *bataua* var. *bataua*.


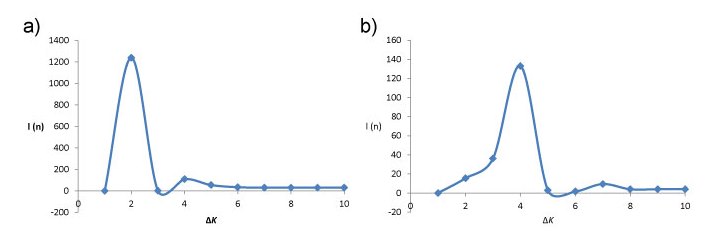


Figure S2. Bar plot of the 644 individuals of *O. bataua* obtained with Structure (Pritchard et al., 2000) using *K* = 2.


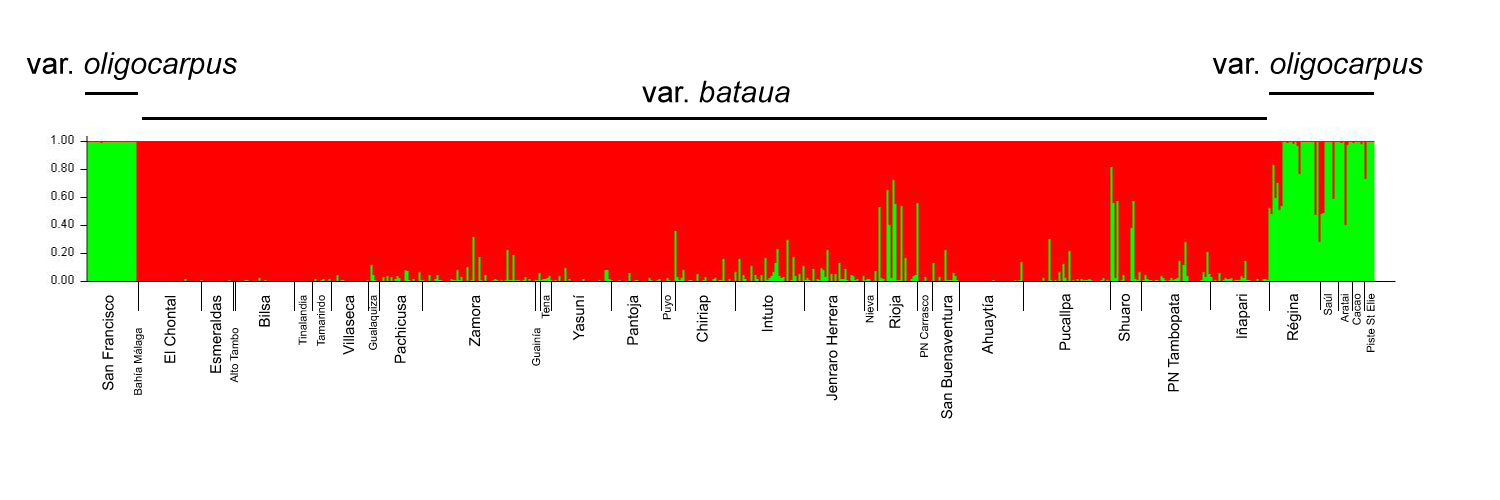


Figure S3. Bar plot of the 566 individuals assigned to the cluster of *O. bataua* var. *bataua* by Structure (Pritchard et al., 2000), obtained with Structure using *K* = 4.

­
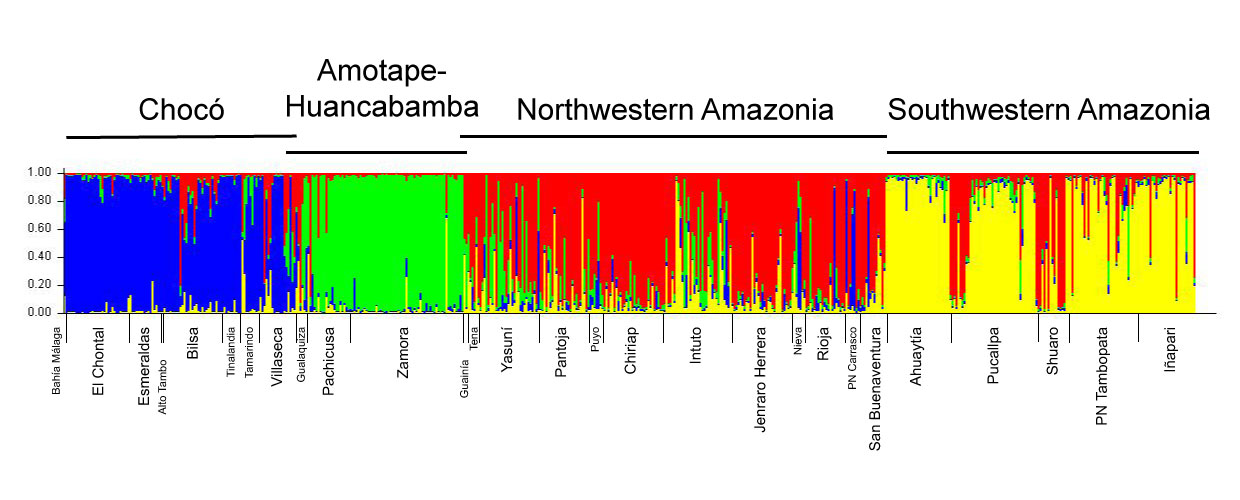


Figure S4. Linear regressions made between: a) Altitude vs. Allelic richness, b) Distance to Intuto (most diverse population) vs. Allelic richness, c) Altitude vs. Allelic richness (without trans-Andean populations), d) Distance to Intuto vs. Allelic richness (without trans-Andean populations). Solid circles represent trans-Andean populations (Esmeraldas, El Chontal, Bilsa, Villaseca). ***** *p* < 0.05


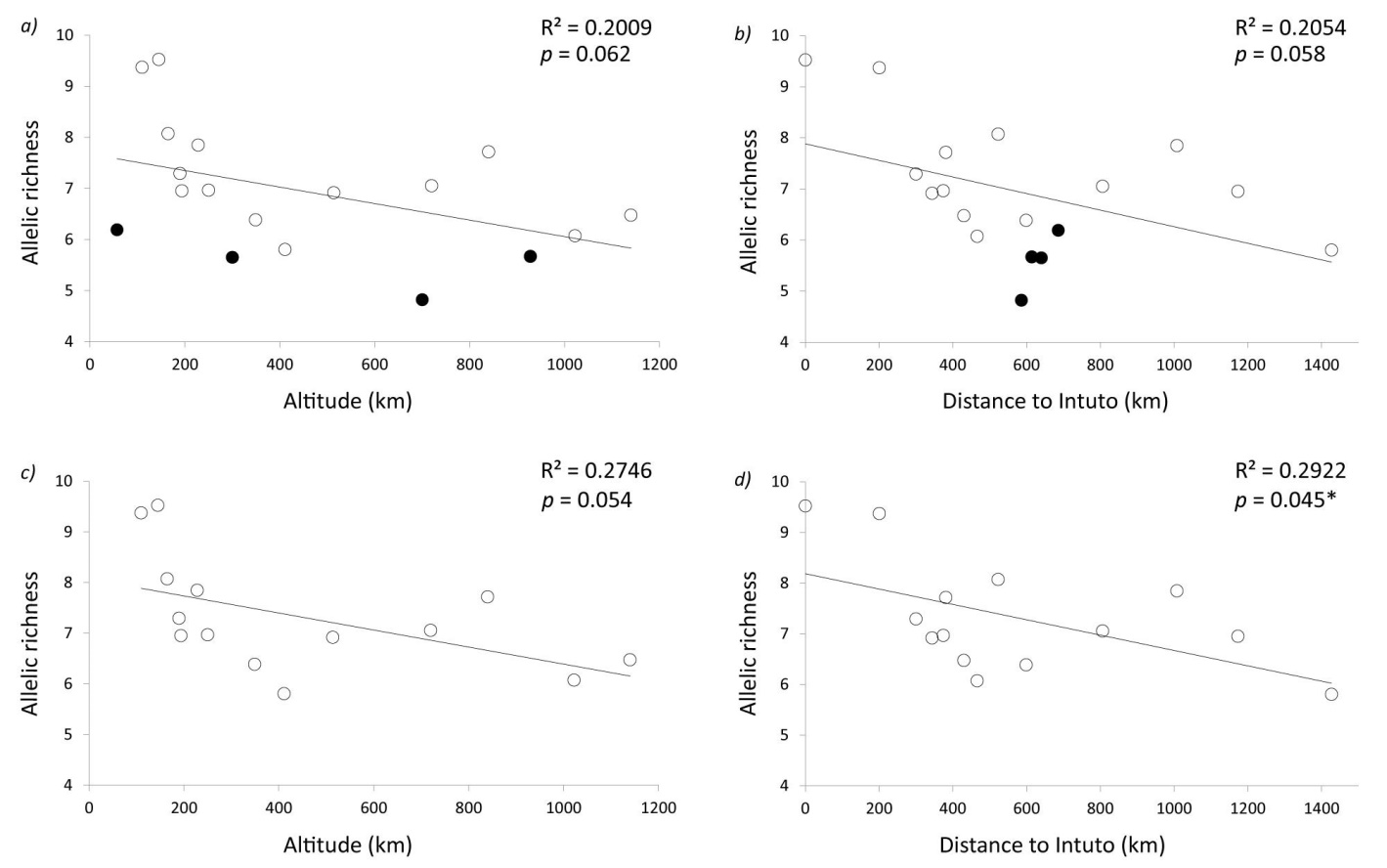

Supplement: Supplementary file 1 [file ECE3-8-8030-s001.docx]
